# Ethnic differences in singleton preterm birth in England and Wales, 2006‐12: Analysis of national routinely collected data

**DOI:** 10.1111/ppe.12585

**Published:** 2019-10-22

**Authors:** Yangmei Li, Maria A. Quigley, Alison Macfarlane, Hiranthi Jayaweera, Jennifer J. Kurinczuk, Jennifer Hollowell

**Affiliations:** ^1^ Policy Research Unit in Maternal Health and Care National Perinatal Epidemiology Unit Nuffield Department of Population Health University of Oxford Oxford UK; ^2^ Centre for Maternal and Child Health Research School of Health Sciences, City University of London London UK; ^3^ School of Anthropology University of Oxford Oxford UK

**Keywords:** ethnic differences, gestational age, health inequalities, mother's country of birth, preterm birth

## Abstract

**Background:**

Data recorded at birth and death registration in England and Wales have been routinely linked with data recorded at birth notification since 2006. These provide scope for detailed analyses on ethnic differences in preterm birth (PTB).

**Objectives:**

We aimed to investigate ethnic differences in PTB and degree of prematurity in England and Wales, taking into account maternal sociodemographic characteristics and to further explore the contribution of mother's country of birth to these ethnic differences in PTB.

**Methods:**

We analysed PTB and degree of prematurity by ethnic group, using routinely collected and linked data for all singleton live births in England and Wales, 2006‐2012. Logistic regression was used to adjust for mother's age, marital status/registration type, area deprivation and mother's country of birth.

**Results:**

In the 4 634 932 births analysed, all minority ethnic groups except ‘Other White’ had significantly higher odds of PTB compared with White British babies (ORs between 1.04‐1.25); highest odds were in Black Caribbean, Indian, Bangladeshi and Pakistani groups. Ethnic differences in PTB tended to be greater at earlier gestational ages. In all ethnic groups, odds of PTB were lower for babies whose mothers were born outside the UK.

**Conclusions:**

In England and Wales, Black Caribbean, Indian, Bangladeshi, Pakistani and Black African babies all have significantly increased odds of being born preterm compared with White British babies. Bangladeshis apart, these groups are particularly at risk of extremely PTB. In all ethnic groups, the odds of PTB are lower for babies whose mothers were born outside the UK. These ethnic differences do not appear to be wholly explained by area deprivation or other sociodemographic characteristics.

**Synopsis**

**Study question**

- What are the ethnic differences in preterm birth in England and Wales, taking into account sociodemographic characteristics?
- How does mother's country of birth contribute to these differences?

**What is already known**

- Risk of preterm birth is high among mothers of Caribbean and West African origin compared with White mothers, whereas the risks in South Asian groups have been less consistent.
- Reasons behind disparities are not well understood.

**What this study adds**

- Black Caribbean, Indian, Bangladeshi, Pakistani and Black African babies all have increased odds of being born preterm compared with White British babies.
- Bangladeshis apart, these groups are particularly at risk of extremely preterm birth.
- Within each group, the odds of preterm birth are lower for babies whose mothers were born outside the UK.

## 1 BACKGROUND

Complications of preterm birth are the largest direct cause of neonatal deaths worldwide, accounting for 35% of 3.1 million neonatal deaths in 2010.^1^ Babies born preterm who survive are also at higher risk of short‐term and long‐term morbidities, including neurodevelopmental impairments and respiratory and gastrointestinal complications, compared with babies born at term.^2^

Much of the research on ethnic differences in preterm birth has been carried out in the United States. This has shown that compared with White mothers, the risks of preterm birth are consistently higher for Black mothers^3^, ^4^, ^5^ but similar or lower for East Asian and Hispanic mothers.^2^, ^4^ Health disparities between ethnic groups have also been observed in England and Wales.^6^, ^7^, ^8^ In particular, the risk of preterm birth, especially very preterm birth, is high among mothers of Caribbean and West African origin compared with White mothers.^9^, ^10^, ^11^, ^12^, ^13^, ^14^, ^15^ South Asian groups have consistently high levels of low birthweight, whereas the risks of preterm birth in South Asian groups compared with the White group have been less consistent.^9^, ^10^, ^11^, ^12^, ^13^, ^14^, ^16^, ^17^, ^18^, ^19^, ^20^ For example, some studies found higher risks,^11^, ^14^, ^16^ some found similar risks^12^, ^13^, ^18^ and others found mixed risks for specific Indian, Pakistani and Bangladeshi groups.^9^, ^10^, ^17^, ^21^ There have been relatively few studies from other high‐income countries.^21^, ^22^, ^23^, ^24^, ^25^, ^26^ Although a number of socio‐economic, obstetric and genetic explanations have been proposed to explain these differences,^1^, ^2^, ^3^, ^16^, ^23^, ^27^, ^28^ the mechanisms or reasons behind them are not well understood.

Previous studies in the United Kingdom (UK) have some limitations. Studies based on regional data have provided important insights but have generally not included all ethnic groups, others have used parents' countries of birth or presented only crude rates because of small numbers.^9^, ^10^, ^11^, ^12^, ^13^, ^14^, ^16^, ^17^, ^18^, ^19^, ^20^, ^29^

National data about gestational age and ethnic group in England and Wales became available after 2005.^10^, ^30^ Data are now available for a longer period so larger numbers of births provide scope for more detailed analyses. The specific objectives of the study were as follows: (a) to investigate ethnic differences in preterm birth and degree of prematurity in England and Wales, taking into account maternal sociodemographic characteristics; and (b) to further explore the contribution of mother's country of birth to these ethnic differences in preterm birth.

## 2 METHODS

### 2.1 Data sources

This was a population‐based study using routinely collected and linked national data on all singleton live births at gestational ages of 22 completed weeks and over in England and Wales between 2006 and 2012 inclusive. De‐identified live birth data for all babies who were born in 2006 through 2012 in England and Wales and those born in this period who died before their first birthday were provided by the Office for National Statistics (ONS) from linked civil registration and birth notification data.^30^

Birth and death registration data are routinely checked by ONS. However, ONS does not routinely exclude all implausible combinations of gestational age and birthweight. We therefore further checked and cleaned the data extract by excluding records with missing values or implausible combinations of gestational age and birthweight by sequentially removed births with: (a) a gestational age greater than or equal to 43 completed weeks; (b) a missing birthweight; (c) an implausible combination of birthweight and gestational age, defined as birthweight more than twice the interquartile range above or below the median birthweight of the sex‐gestation‐ethnic group‐specific stratum of the study dataset.

### 2.2 Ethnic group and other explanatory variables

Staff notifying a baby's birth to the birth notification system are asked to record the baby's ethnic group as reported by the mother based on the ethnic categories used in the 2001 Census in England and Wales.^10^ These ethnic categories are based on three most common self‐defined groups in the UK (‘White’, ‘Asian’ and ‘Black’) with several country or regional categories within each of them, a ‘Mixed’ group with several Mixed background categories, and a ‘Chinese or other’ group.^31^

These ethnic group categories were then recoded for analysis into White British, Other White, three Asian or Asian British groups (Indian, Pakistani and Bangladeshi), two Black or Black British groups (Black Caribbean and Black African), a ‘Mixed/Other’ group, which included all Mixed groups, Other Asian, Other Black, Chinese and Other, and a ‘Not stated’ group. The above individual minority ethnic groups were selected and included for analysis because they were the most common minority ethnic groups in England and Wales. All Mixed groups and ‘Other’ groups were aggregated because of small numbers and the complexity of the heterogeneous composition of the subgroups which would have made any results difficult to interpret. Details of the derivation of the ethnic group categories are reported elsewhere.^6^

Other explanatory variables included the baby's sex and year of birth, age of mother, area deprivation score of the mother's area of residence as coded according to the 2015 English Index of Multiple Deprivation (IMD)^32^ and the 2014 Welsh IMD,^33^ mother's country of birth, parents' marital status/registration type and mother's country of residence.

### 2.3 Outcome measures

The primary outcome was preterm birth, defined as the live birth of a baby at less than 37 completed weeks of gestation. Our outcome measure was the percentage of live births that were preterm. A secondary outcome was degree of prematurity: (a) late and moderately preterm births (32‐36 completed weeks); (b) very preterm births (28‐31 completed weeks); and (c) extremely preterm births (22‐27 completed weeks).

### 2.4 Statistical analysis

Logistic regression models were used to explore ethnic differences in preterm birth using the White British group as the reference group. We adjusted all models for the baby's sex and year of birth to account for differences between sexes and over time (models adjusted for these two variables only are referred to as the ‘base model’ hereafter). Further adjustments were made (referred to as ‘adjusted model’) to account for IMD quintiles, age of mother (under 18, 18‐19, 20‐24, 25‐29, 30‐34, 35‐39, 40 and over) and parents' marital status/registration type (married, joint registration/same address, joint registration/ different address, sole registration). As IMD scores are constructed differently in England and in Wales, we adjusted for the mother's country of residence (England vs Wales) in all models that included IMD.

Multinomial logistic regression was used to investigate the association between ethnic group and degree of prematurity, treating degree of prematurity as a categorical outcome variable and adjusting for the same covariates as above.

To further explore the contribution of mother's country of birth to ethnic differences in the odds of preterm birth, we then included mother's country of birth (inside versus outside the UK) in the adjusted model, tested the interaction between ethnic group and mother's country of birth, and present stratified results as appropriate. Finally, to assist in the interpretation of findings, we carried out additional exploratory analyses in which we adjusted for covariates (mother's country of birth, IMD quintiles, age of mother and parents' marital status/registration type) individually. All analyses were conducted in STATA version 13.

### 2.5 Ethics approval

The study was approved by ‘National Research Ethics Service (NRES) Committee South Central—Oxford B’ (Research Ethics Committee reference number: 15/SC/0493).

## 3 RESULTS

The original dataset included 4 744 666 singleton live births at gestational ages of 22 weeks or more in England and Wales from 2006 to 2012. We sequentially excluded 16 695 births with implausible gestational age, 20 999 with missing birthweight and 72 040 with implausible sex‐gestation‐ethnic group‐specific birthweight, adding up to 109 734 (2.3%) births excluded in total. The study population therefore consisted of 4 634 932 singleton live‐born babies at gestational ages of 22‐42 completed weeks. Around 65% of the study population was White British, 7% was Other White, 8% was South Asian (consisting of Indian, Pakistani and Bangladeshi), 4% was Black (consisting of Black Caribbean and Black African), 9% was in the ‘Mixed/Other’ group. The remaining 6% of the study population had an ethnic group ‘not stated’.

### 3.1 Characteristics of the mothers and babies

Ethnicity was strongly associated with characteristics of mothers and babies (Table 1). South Asian, Other White and Black African babies were less likely to be born to mothers aged under 20 compared with White British babies, while Black Caribbean babies were more likely to be born to this group of young mothers. South Asian babies were less likely to be born to mothers aged 35 and over compared with White British babies, while Other White, Black Caribbean and Black African babies were equally or slightly more likely to be born to this group of older mothers.

**Table 1:** Characteristics of the study population and preterm birth rates by ethnic group (percentage, singleton live births, England and Wales, 2006‐2012)

Ethnic group (n = 4 634 932) (Total number)	White British (3 009 231)	Other White (340 526)	Indian (132 651)	Pakistani (180 269)	Bangladeshi (62 948)	Black Caribbean (47 505)	Black African (154 076)	Mixed/ Other (419 970)	Not stated (287 756)
Percentage	64.9	7.4	2.9	3.9	1.4	1.0	3.3	9.1	6.2
Age of mother (y)									
<20	7.1	2.6	0.7	2.0	2.2	8.5	2.6	5.2	5.8
20‐34	73.0	76.5	84.4	85.8	87.1	69.4	74.6	73.9	73.1
≥35	19.9	20.9	14.9	12.3	10.7	22.1	22.9	20.9	21.0
Deprivation quintile									
1 (most deprived)	23.4	23.8	24.3	55.3	59.8	49.8	50.1	34.2	22.0
2	20.2	26.1	29.9	25.1	24.8	29.9	29.6	25.9	23.1
3	19.5	20.0	20.8	10.8	8.8	13.0	11.9	17.2	20.1
4	18.9	16.2	13.6	5.4	4.3	4.8	5.4	12.5	18.6
5 (least deprived)	18.0	14.0	11.4	3.4	2.3	2.4	3.1	10.3	16.2
Mother's country of birth^a^									
Non‐UK	3.9	80.1	66.0	62.9	78.2	36.7	92.7	59.6	26.4
Marital status/registration type									
Married	46.2	64.8	96.2	95.7	94.6	26.5	61.3	61.5	57.1
Joint registration/same address	36.7	26.8	2.1	1.9	2.7	21.7	14.6	19.1	28.6
Joint registration/different address	10.9	4.0	0.9	1.1	1.6	32.0	12.4	11.2	8.3
Sole registration	6.2	4.4	0.9	1.3	1.2	19.8	11.8	8.2	6.1
Gestational age (wk)									
22‐27	0.26	0.23	0.31	0.38	0.29	0.90	0.70	0.36	0.35
28‐31	0.56	0.42	0.62	0.67	0.54	1.13	0.87	0.59	0.59
32‐33	0.68	0.52	0.72	0.70	0.74	0.98	0.79	0.68	0.70
34‐36	4.03	3.46	4.36	4.25	4.73	5.20	3.82	4.01	4.00
37‐42	94.47	95.37	93.99	94.00	93.70	91.79	93.82	94.36	94.36
Preterm birth (22‐36 wk)	5.53	4.63	6.01	6.00	6.30	8.21	6.18	5.64	5.64

a Percentages based on study population excluding records with missing data on mother's country of birth (n = 143 in total).

Pakistani, Bangladeshi, Black Caribbean and Black African babies were more likely to be born to mothers living in the most deprived areas (between 50%‐60% in IMD quintile 1 and approximately 80% in IMD quintiles 1 and 2 combined) compared with White British babies (23% and 44%, respectively).

The majority of White British (96.1%) and Black Caribbean (63.3%) babies were born to mothers who themselves were born in the UK, while the majority of babies in the other ethnic groups were born to mothers born outside the UK, ranging from 62.9% in the Pakistani group to 92.7% in Black African babies. This reflects differences in migration histories and timescales among ethnic groups in England and Wales.

Compared with parents of White British babies, parents of South Asian and Other White babies were more likely to be married or jointly registered as living at the same address while parents of Black babies, especially Black Caribbean babies, were less likely to be married or jointly registered as living at the same address.

The absolute risk of preterm birth was also strongly associated with these characteristics, having decreased over time and plateaued in 2010. The risk was highest in male babies, in the youngest and oldest groups of mothers, those living in the most deprived areas, mothers born in the UK and mothers who were sole registrants or jointly registered as living at different addresses from the baby's father; these results were confirmed using adjusted odds ratios (Table S1).

### 3.2 Associations between ethnic group and preterm birth

Overall, preterm births accounted for 5.6% (n = 258 515) of the whole study population. Preterm birth rates were lowest among White babies, with 5.5% of White British babies and 4.6% of Other White babies being born preterm (Table 1). Black Caribbean babies had the highest rate at 8.2%. Rates for the three South Asian groups and the Black African group were similar, ranging from 6.0% for Pakistani to 6.3% for Bangladeshi babies.

**Figure 1:**
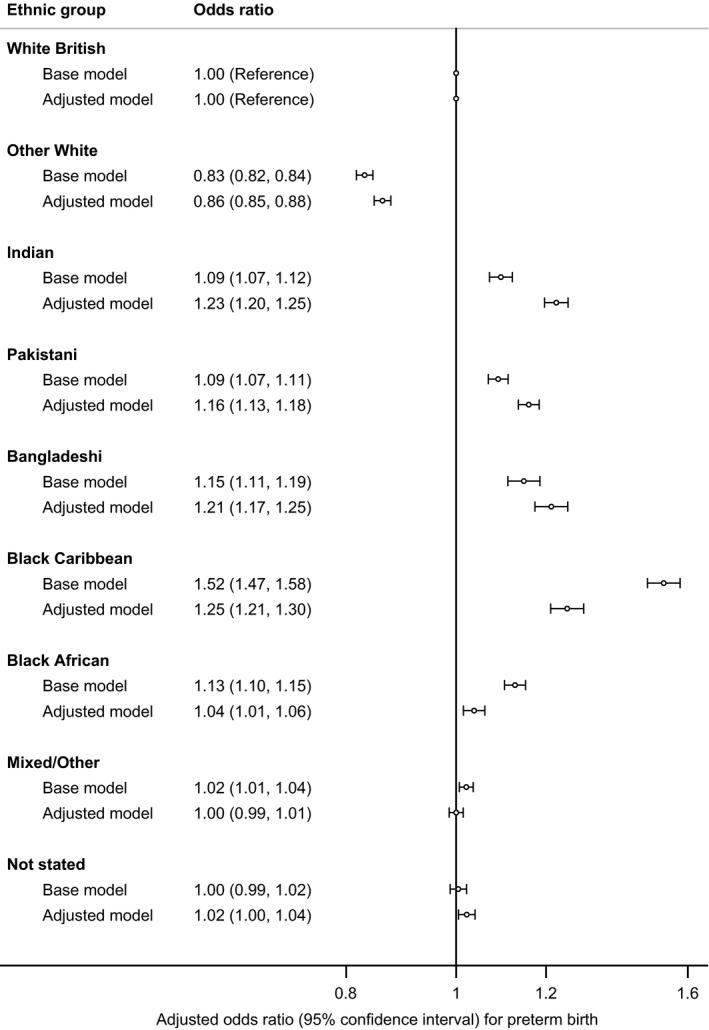
Preterm birth by ethnic group (singleton live births, England and Wales, 2006‐2012). Base model adjusted for baby's sex and year of birth; adjusted model adjusted for variables in Base model and additionally adjusted for age of mother, deprivation quintile and marital status/registration type

Figure 1 shows adjusted odds ratios (OR) for preterm birth by ethnic group, using White British babies as the reference group, with first the base model adjusted only for the baby's sex and year of birth and then with further adjustment for age of mother, deprivation quintile and parents' marital status/registration type.

In the base model, the lowest odds were for Other White babies which were below that for White British babies (OR 0.83, 95% CI 0.82, 0.84). For all other groups, the odds of preterm birth were higher than for White British babies with ORs ranging from 1.09 for Indian and Pakistani babies to 1.52 (95% CI 1.47, 1.58) for Black Caribbean babies.

After further adjustment, the relationships were broadly the same, but the adjusted ORs for Indians, Pakistanis and Bangladeshis increased in magnitude while the adjusted OR for Black Caribbean and Black African babies decreased in magnitude. Adjustment increased the odds in Other White babies, but odds in this group remained the lowest (OR 0.86, 95% CI 0.85, 0.88). Amongst the groups with increased odds, adjusted ORs ranged from 1.04 (95% CI 1.01, 1.06) for Black African babies to 1.25 (95% CI 1.21, 1.30) for Black Caribbean, 1.23 (95% CI 1.20, 1.25) for Indian and 1.21 (95% CI 1.17, 1.25) for Bangladeshi babies.

### 3.3 Ethnic group and degree of prematurity

Compared with White babies, babies of other ethnic backgrounds were generally more likely to be born at earlier gestational ages. Rates of extremely preterm birth were particularly high among babies of Black Caribbean and Black African origin (Table 1).

**Figure 2:**
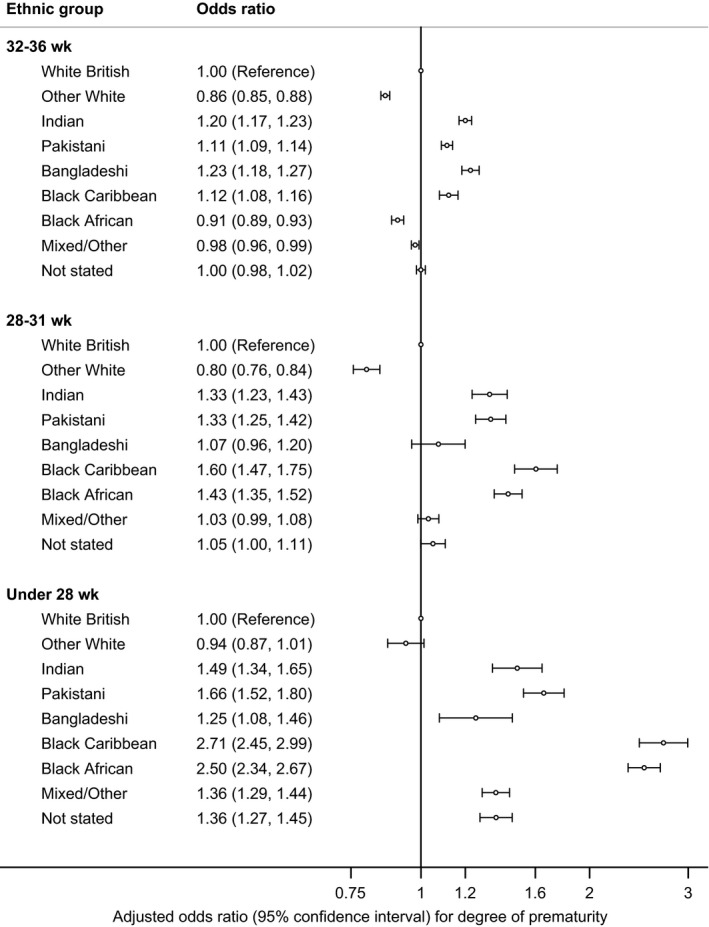
The association between ethnic group and degree of prematurity (singleton live births, England and Wales, 2006‐2012). Odds ratios are adjusted for baby's sex, baby's year of birth, age of mother, deprivation quintile and marital status/registration type

In the adjusted multinomial model, the differences compared with the White British group were greater at earlier gestational ages for all groups except Other White and Bangladeshi groups (Figure 2). The differences were most marked among Black African and Black Caribbean babies for whom the ORs were around 1.5 for being born at 28‐31 weeks and increased to around 2.6 for being born at 22‐27 weeks. The differences were less marked for Indian and Pakistani babies.

### 3.4 Baby's ethnic group, mother's country of birth and preterm birth

**Figure 3:**
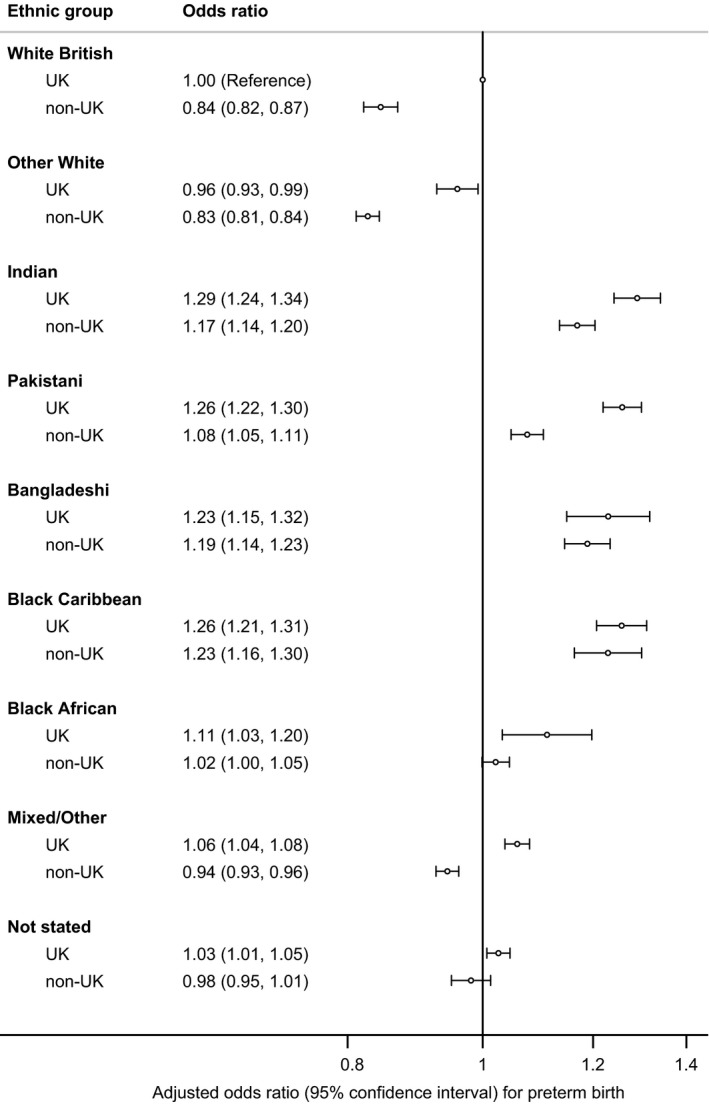
The association between ethnic group, mother's country of birth and preterm birth (singleton live births, England and Wales, 2006‐2012). Odds ratios are adjusted for baby's sex, baby's year of birth, age of mother, deprivation quintile and marital status/registration type

As we found strong interaction (*P* < .001) between ethnic group and whether the mother's country of birth was inside or outside the UK, we further stratified the study population into 18 subcategories by ethnic group (9 groups) and mother's country of birth, inside or outside UK, and compared the odds of preterm birth using White British babies born to mothers born in the UK as the reference group. Figure 3 and Table S2 show that consistently, within ethnic group, UK‐born mothers had higher odds of preterm birth than non‐UK‐born mothers. The ‘univariable’ analysis adjusting for baby's sex, year of birth and each covariate individually also show that for all ethnic groups except Other White, adjusting for mother's country of birth increases the magnitude of the association between ethnicity and preterm birth (Table S3). Exploratory analysis describing the characteristics of the study population by ethnic group and mother's country of birth (Table S4) shows that for ethnic minority groups, in general, although non‐UK‐born mothers were more likely to live in the more deprived areas compared with UK‐born mothers, they were also less likely to be younger mothers and more likely to be married or live at the same address with their partners.

## 4 COMMENT

### 4.1 Principal findings

Analysis of a national linked dataset of over 4.6 million singleton live‐born babies shows that ethnic differences exist in preterm birth in England and Wales. Black Caribbean babies have the highest crude preterm birth rate, followed by Bangladeshi, Black African, Indian and Pakistani babies. Maternal sociodemographic characteristics do not appear to explain the higher odds of preterm birth among minority ethnic groups (except Other White) compared with White British births. After adjustment, the highest odds are found in Black Caribbean and South Asian groups. Ethnic differences in preterm birth tend to be greater at earlier gestational ages. In all ethnic groups, the odds of preterm birth are lower for babies whose mothers were born outside the UK.

### 4.2 Strengths of the study

The complete coverage of birth registration data, the large number of births and the low proportion with missing data mean that selection bias, detection and observer bias are minimized. The dataset also included sufficient numbers of births in the smaller ethnic groups, such as Black Caribbean and Bangladeshi groups, to enable disaggregated analyses.

### 4.3 Limitations of the data

There is limited information available about the quality of ethnicity recording although it is possible that in some cases the mother may record her own ethnic group or that health care staff may record the mother or baby's ethnic group rather than asking the mother to report it.^10^ Although some misclassification of ethnic group is possible, previous analysis suggests that this may predominantly affect the Mixed ethnic group^10^ which was not the focus of our study. The availability of data about mother's country of birth linked to data about ethnicity enabled us to study country of birth and ethnicity separately, and this is a strength of our analysis.

We lack data on how gestational age was measured and the accuracy of the measurement, but the birth notification data that we used are regarded as the best source for national data on gestational age.^34^ Birth attendants are asked to record data about gestational age based on last menstrual period in the birth notification system, but it seems probable that they were taken directly from woman's medical records and in the majority of cases, this would have been based on more accurate ultrasound dating. Information about clinical subtypes of preterm birth, that is spontaneous, preterm premature rupture of membranes and medically indicated (elective), is not available from the registration and birth notification systems. We also lack data about pregnancy history and other socio‐economic or lifestyle factors that may be related to preterm birth^2^ and differ between ethnic groups. IMD is based on the baby/mother's postcode of usual residence and may not capture the exposure during pregnancy for all women. However, IMD is an area‐based measure of deprivation that is widely used in health research despite its limitations^35^ and was used for this particular study because individual socio‐economic status is available for only a 10% sample of births whereas IMD provides a measure of socio‐economic disadvantage available for all births.

Although these national‐level results are applicable to the populations of England and Wales, the different compositions of ethnic groups in other countries may limit international generalisability. In particular, we have used a combined ‘Black African’ group which consists of a diverse range of people originating from a whole continent within which there are wide differences in risks of preterm birth.^15^ The generalisability to other parts of the world may also be affected by differences in health care access.

### 4.4 Interpretation

Findings of higher odds of preterm birth in the Black groups are consistent with previous studies.^9^, ^10^, ^11^, ^12^, ^13^, ^14^, ^15^ Area deprivation and marital status have been suggested to explain half of the excess risk of preterm birth in Afro‐Caribbean but not African mothers.^12^ We similarly found that maternal sociodemographic characteristics only accounted for part of the higher odds of preterm birth in Black Caribbean and Black African babies. Raised risks of preterm birth have also been associated with bacterial vaginosis^36^ which is more common in Black women compared with White women.^28^, ^36^ This difference might account for approximately 30%, or even up to 60% of the excess preterm births in the Black groups.^28^ Socio‐economic status, stress, substance abuse, racism, mother's size, biological variations in gestation, previous preterm history, birth interval and access to health care have all been proposed as potential explanations for preterm birth.^1^, ^2^, ^3^, ^16^, ^23^, ^27^, ^28^ Universal access to health care in England and Wales should mean that all ethnic groups are able to access antenatal care, but in practice, some ethnic minority women may experience barriers affecting the antenatal care that they receive.^37^

Higher odds of preterm birth identified in all the three South Asian (Indian, Pakistani and Bangladeshi) groups in our study have been less well established.^9^, ^10^, ^11^, ^12^, ^13^, ^14^, ^16^, ^17^, ^18^, ^19^, ^20^, ^21^, ^24^ Previous studies in which South Asian groups were aggregated showed inconsistent results with some showing higher risks^11^, ^14^, ^16^, ^24^ and others finding no increase in risks.^12^, ^13^, ^18^ However, as South Asian groups are heterogeneous, results based on aggregated analysis may be misleading. In studies that disaggregated South Asian groups, higher risks of preterm birth were sometimes only found in Indian^9^, ^17^ and/or Pakistani groups^10^, ^21^ but not all groups.^9^, ^10^, ^17^, ^21^ These disaggregated analyses typically have used groups with smaller numbers than ours and some differ from the national population in their ethnic composition.

It is plausible that higher odds of preterm birth in the South Asian groups are due to a higher prevalence of medical conditions that are associated with medically indicated preterm birth in these groups, but this is not something we were able to investigate. A UK study found being South Asian was a risk factor for medically indicated but not spontaneous delivery before 34 weeks.^18^ Shorter gestational age and a non‐significant increase in the risk of preterm birth have also been observed in Indo‐Asian women in England compared with White British among women with chronic hypertension.^29^ It is also plausible that South Asian fetuses, similar to Black fetuses, mature earlier in general compared with White fetuses.^11^, ^16^

Our finding that ethnic differences in the odds of preterm birth tend to be greater at earlier gestational ages has been less well studied as most studies only use one cut‐off for preterm birth. However, our results are broadly consistent with findings from the few studies that have used different cut‐off points for preterm birth to explore the changes in ethnic variation for Black groups.^12^, ^38^, ^39^, ^40^, ^41^ In particular, a study investigating North Birmingham women delivering singletons also showed that in Afro‐Caribbean and African women, the risk of preterm birth was particularly high for delivery before 34 and 28 weeks.^12^ However, the risks in South Asian women compared with White groups were not higher at earlier gestational ages in those studies.^12^, ^40^

We cannot provide an explanation of the ethnic differences in the increased odds of preterm birth at earlier gestational ages. It is worth exploring this phenomenon and potential mechanisms in other studies as very preterm births (births before 32 weeks’ gestational age) account for the majority of neonatal deaths and serious morbidity.^27^

Another important finding of our study is that within each ethnic group, babies of non‐UK‐born mothers had lower odds of preterm birth than those of UK‐born mothers. The implications of this are likely to vary as the proportion of minority ethnic mothers born outside the UK varies. Differences in mother's country of birth by ethnic group, as seen in our study, may reflect differences in migration history, length of residence in the host country and degree of acculturation.^42^ However, according to our exploratory analysis, in general, although non‐UK‐born mothers were more likely to live in the more deprived areas compared with UK‐born mothers, which in turn would increase their odds of preterm birth, they were also less likely to be younger mothers and more likely to be married or live at the same address with their partners which are associated with lower odds for preterm birth. The finding of lower odds of preterm birth in non‐UK‐born mothers is broadly consistent with results from other studies that showed foreign‐born mothers in ethnic minority groups had similar or lower risks of preterm birth compared with their US‐born or UK‐born counterparts.^15^, ^22^, ^43^, ^44^ The healthy migrant effect and the further loss of this effect after one generation have been suggested as a potential explanation.^5^ Evidence also suggests that being able to maintain cultural links or live in areas with higher proportion of people from the same ethnic group may outweigh other hurdles and contribute to better well‐being in general or pregnancy outcomes in particular in ethnic minority groups.^45^, ^46^ Unfortunately, we were unable to further explore these factors because of a lack of relevant data in our dataset.

## 5 CONCLUSIONS

In England and Wales, Black Caribbean, Indian, Bangladeshi, Pakistani and Black African babies all have increased odds of being born preterm compared with White British babies. Bangladeshis apart, these groups are particularly at risk of extremely preterm birth. In all ethnic groups, the odds of preterm birth are lower for babies whose mothers were born outside the UK. These ethnic differences do not appear to be wholly explained by area deprivation or other sociodemographic characteristics; therefore, other factors should be explored, including the causes and subtypes of preterm birth, particularly the contribution of medically indicated preterm birth. The higher odds of preterm birth observed in UK‐born compared with non‐UK‐born mothers in all ethnic groups may point to risk factors that increase with acculturation.

## References

[1] Blencowe H , Cousens S , Chou D , et al. Born too soon: the global epidemiology of 15 million preterm births. Reprod Health. 2013;10(suppl 1):S2. 24625129 10.1186/1742-4755-10-S1-S2 PMC3828585

[2] Goldenberg RL , Culhane JF , Iams JD , Romero R . Epidemiology and causes of preterm birth. Lancet. 2008;371:75‐84. 18177778 10.1016/S0140-6736(08)60074-4 PMC7134569

[3] Manuck TA . Racial and ethnic differences in preterm birth: a complex, multifactorial problem. Semin Perinatol. 2017;41:511‐518. 28941962 10.1053/j.semperi.2017.08.010 PMC6381592

[4] Schaaf JM , Liem SM , Mol BW , Abu‐Hanna A , Ravelli AC . Ethnic and racial disparities in the risk of preterm birth: a systematic review and meta‐analysis. Am J Perinatol. 2013;30:433‐450. 23059494 10.1055/s-0032-1326988

[5] Sorbye IK , Wanigaratne S , Urquia ML . Variations in gestational length and preterm delivery by race, ethnicity and migration. Best Pract Res Clin Obstet Gynaecol. 2016;32:60‐68. 26458997 10.1016/j.bpobgyn.2015.08.017

[6] Li Y , Quigley MA , Dattani N , et al. The contribution of gestational age, area deprivation and mother's country of birth to ethnic variations in infant mortality in England and Wales: a national cohort study using routinely collected data. PLoS ONE. 2018;13:e0195146. 29649290 10.1371/journal.pone.0195146 PMC5896919

[7] Wohland P , Rees P , Nazroo J , Jagger C . Inequalities in healthy life expectancy between ethnic groups in England and Wales in 2001. Ethn Health. 2015;20:341‐353. 24897306 10.1080/13557858.2014.921892 PMC4648377

[8] Hollowell J , Kurinczuk JJ , Brocklehurst P , Gray R . Social and ethnic inequalities in infant mortality: a perspective from the United kingdom. Semin Perinatol. 2011;35:240‐244. 21798404 10.1053/j.semperi.2011.02.021

[9] Kelly Y , Panico L , Bartley M , Marmot M , Nazroo J , Sacker A . Why does birthweight vary among ethnic groups in the UK? Findings from the millennium cohort study. J Public Health (Oxf). 2009;31:131‐137. 18647751 10.1093/pubmed/fdn057

[10] Moser K , Stanfield KM , Leon DA . Birthweight and gestational age by ethnic group, England and Wales 2005: introducing new data on births. Health Stat Q. 2008;22–31:34‐55. 18810886

[11] Patel RR , Steer P , Doyle P , Little MP , Elliott P . Does gestation vary by ethnic group? A London‐based study of over 122,000 pregnancies with spontaneous onset of labour. Int J Epidemiol. 2004;33:107‐113. 15075154 10.1093/ije/dyg238

[12] Aveyard P , Cheng KK , Manaseki S , Gardosi J . The risk of preterm delivery in women from different ethnic groups. BJOG. 2002;109:894‐899. 12197368 10.1111/j.1471-0528.2002.01197.x

[13] Lyon AJ , Clarkson P , Jeffrey I , West GA . Effect of ethnic origin of mother on fetal outcome. Arch Dis Child Fetal Neonatal Ed. 1994;70:F40‐43. 8117126 10.1136/fn.70.1.f40 PMC1060986

[14] Steer P , Alam MA , Wadsworth J , Welch A . Relation between maternal haemoglobin concentration and birth weight in different ethnic groups. BMJ. 1995;310:489‐491. 7888886 10.1136/bmj.310.6978.489 PMC2548871

[15] Datta‐Nemdharry P , Dattani N , Macfarlane AJ . Birth outcomes for African and Caribbean babies in England and Wales: retrospective analysis of routinely collected data. BMJ Open. 2012;2(3):e001088. 10.1136/bmjopen-2012-001088 PMC3364453 22619268

[16] Balchin I , Steer PJ . Race, prematurity and immaturity. Early Hum Dev. 2007;83:749‐754. 17928174 10.1016/j.earlhumdev.2007.09.003

[17] Garcia R , Ali N , Guppy A , Griffiths M , Randhawa G . Differences in the pregnancy gestation period and mean birth weights in infants born to Indian, Pakistani, Bangladeshi and white British mothers in Luton, UK: a retrospective analysis of routinely collected data. BMJ Open. 2017;7:e017139. 10.1136/bmjopen-2017-017139 PMC5724131 28801435

[18] Khalil A , Rezende J , Akolekar R , Syngelaki A , Nicolaides KH . Maternal racial origin and adverse pregnancy outcome: a cohort study. Ultrasound Obstet Gynecol. 2013;41:278‐285. 23023978 10.1002/uog.12313

[19] Stacey T , Prady S , Haith‐Cooper M , Downe S , Simpson N , Pickett K . Ethno‐specific risk factors for adverse pregnancy outcomes: findings from the born in bradford cohort study. Matern Child Health J. 2016;20:1394‐1404. 26983444 10.1007/s10995-016-1936-x PMC4909785

[20] Versi E , Liu KL , Chia P , Seddon G . Obstetric outcome of Bangladeshi women in east London. Br J Obstet Gynaecol. 1995;102:630‐637. 7654641 10.1111/j.1471-0528.1995.tb11401.x

[21] Bansal N , Chalmers JW , Fischbacher CM , et al. Ethnicity and first birth: age, smoking, delivery, gestation, weight and feeding: Scottish health and ethnicity linkage study. Eur J Public Health. 2014;24:911‐916. 24843052 10.1093/eurpub/cku059 PMC4140759

[22] Urquia ML , Glazier RH , Blondel B , et al. International migration and adverse birth outcomes: role of ethnicity, region of origin and destination. J Epidemiol Community Health. 2010;64:243‐251. 19692737 10.1136/jech.2008.083535 PMC2922721

[23] Bollini P , Pampallona S , Wanner P , Kupelnick B . Pregnancy outcome of migrant women and integration policy: a systematic review of the international literature. Soc Sci Med. 2009;68:452‐461. 19042065 10.1016/j.socscimed.2008.10.018

[24] Malin M , Gissler M . Maternal care and birth outcomes among ethnic minority women in Finland. BMC Public Health. 2009;9:84. 19298682 10.1186/1471-2458-9-84 PMC2674879

[25] Goedhart G , van Eijsden M , van der Wal MF , Bonsel GJ . Ethnic differences in preterm birth and its subtypes: the effect of a cumulative risk profile. BJOG. 2008;115:710‐719. 18410654 10.1111/j.1471-0528.2008.01682.x

[26] Khanolkar AR , Wedren S , Essen B , Sparen P , Koupil I . Preterm and postterm birth in immigrant‐ and Swedish‐born parents: a population register‐based study. Eur J Epidemiol. 2015;30:435‐447. 25687167 10.1007/s10654-014-9986-0

[27] Kramer MR , Hogue CR . What causes racial disparities in very preterm birth? A biosocial perspective. Epidemiol Rev. 2009;31:84‐98. 19477907 10.1093/ajerev/mxp003 PMC4361938

[28] Fiscella K . Racial disparities in preterm births. The role of urogenital infections. Public Health Rep. 1996;111:104‐113. 8606905 PMC1381713

[29] Lydakis C , Beevers DG , Beevers M , Lip GY . Obstetric and neonatal outcome following chronic hypertension in pregnancy among different ethnic groups. QJM. 1998;91:837‐844. 10024949 10.1093/qjmed/91.12.837

[30] Hilder L , Moser K , Dattani N , Macfarlane A . Pilot linkage of NHS numbers for babies data with birth registrations. Health Stat Q. 2007;25‐33. 17373380

[31] Simpson L , Jivraj S , Warren J . The stability of ethnic identity in England and Wales 2001–2011. J R Stat Soc Ser A Stat Soc. 2016;179:1025‐1049. 10.1111/rssa.12175 PMC5053233 27773972

[32] Smith T , Noble M , Noble S , Wright G , McLennan D , Plunkett E .The English indices of deprivation 2015 technical report: department for communities and local government 2015.

[33] Welsh index of multiple deprivation (WIMD) 2014 revised: Welsh government 2014.

[34] Ghosh RE , Ashworth DC , Hansell AL , Garwood K , Elliott P , Toledano MB . Routinely collected English birth data sets: comparisons and recommendations for reproductive epidemiology. Arch Dis Child. 2016;101:F451‐457. 10.1136/archdischild-2015-309540 26837309

[35] Fieldhouse EA , Tye R . Deprived people or deprived places? Exploring the ecological fallacy in studies of deprivation with the samples of anonymised records. Environ Planning A. 1996;28:237‐259.

[36] Hay PE , Lamont RF , Taylor‐Robinson D , Morgan DJ , Ison C , Pearson J . Abnormal bacterial colonisation of the genital tract and subsequent preterm delivery and late miscarriage. BMJ. 1994;308:295‐298. 8124116 10.1136/bmj.308.6924.295 PMC2539287

[37] Henderson J , Gao H , Redshaw M . Experiencing maternity care: the care received and perceptions of women from different ethnic groups. BMC Pregnancy Childbirth. 2013;13:196. 24148317 10.1186/1471-2393-13-196 PMC3854085

[38] McKinnon B , Yang S , Kramer MS , Bushnik T , Sheppard AJ , Kaufman JS . Comparison of black‐white disparities in preterm birth between Canada and the United States. CMAJ. 2016;188:E19‐26. 26553860 10.1503/cmaj.150464 PMC4695373

[39] Carmichael SL , Kan P , Padula AM , et al. Social disadvantage and the black‐white disparity in spontaneous preterm delivery among California births. PLoS ONE. 2017;12:e0182862. 28800643 10.1371/journal.pone.0182862 PMC5553771

[40] Shiono PH , Klebanoff MA . Ethnic differences in preterm and very preterm delivery. Am J Public Health. 1986;76:1317‐1321. 3766827 10.2105/ajph.76.11.1317 PMC1646746

[41] Zhang J , Savitz DA . Preterm birth subtypes among blacks and whites. Epidemiology. 1992;3:428‐433. 1391135 10.1097/00001648-199209000-00008

[42] Dustmann C , Frattini T , Theodoropoulos N . Ethnicity and second generation immigrants in Britain. CReAM Discussion Paper Series 1004: Centre for Research and Analysis of Migration (CReAM) DoE, University College London 2010.

[43] Elo IT , Vang Z , Culhane JF . Variation in birth outcomes by mother's country of birth among non‐Hispanic black women in the United States. Matern Child Health J. 2014;18:2371‐2381. 24756226 10.1007/s10995-014-1477-0 PMC4207849

[44] Singh GK , Yu SM . Adverse pregnancy outcomes: differences between US‐ and foreign‐born women in major US racial and ethnic groups. Am J Public Health. 1996;86:837‐843. 8659659 10.2105/ajph.86.6.837 PMC1380404

[45] Dorsett R , Rienzo C , Weale M . Intergenerational and inter‐ethnic well‐being: an analysis for the UK. National Institute of Economic and Social Research (NIESR) Discussion Papers 451: National Institute of Economic and Social Research 2015.

[46] Pickett KE , Collins Jr JW , Masi CM , Wilkinson RG . The effects of racial density and income incongruity on pregnancy outcomes. Soc Sci Med. 2005;60:2229‐2238. 15748671 10.1016/j.socscimed.2004.10.023

