# Supplementary material for: Ethnic differences in singleton preterm birth in England and Wales, 2006‐12: Analysis of national routinely collected data
Source: Paediatr Perinat Epidemiol. 2019 Oct 22;33(6):449–58. doi: 10.1111/ppe.12585 (PMC6900067; doi:10.1111/ppe.12585)
Supplement: Supplementary file 1 [file PPE-33-449-s001.docx]

**Table S1 Preterm birth by mother’s and baby’s characteristics (singleton live births, England and Wales, 2006-2012)**

| **Characteristics** | **Preterm births** | **Total live births** | **Preterm birth rate** | **Base model^a^** | **Adjusted model^b^** |
| --- | --- | --- | --- | --- | --- |
|  |  |  | **% (95% CI)** | **OR (95% CI)** | **OR (95% CI)** |
| **All** | 258,515 | 4,634,932 | 5.58 (5.56, 5.60) | - | - |
| **Baby’s sex** |  |  |  |  |  |
| Male | 140,994 | 2,377,766 | 5.93 (5.90, 5.96) | 1.00 (Reference) | 1.00 (Reference) |
| Female | 117,521 | 2,257,166 | 5.21 (5.18, 5.24) | 0.87 (0.86, 0.88) | 0.87 (0.86, 0.88) |
| **Baby's year of birth** |  |  |  |  |  |
| 2006 | 37,858 | 631,705 | 5.99 (5.93, 6.05) | 1.00 (Reference) | 1.00 (Reference) |
| 2007 | 37,184 | 646,902 | 5.75 (5.69, 5.80) | 0.96 (0.94, 0.97) | 0.96 (0.94, 0.97) |
| 2008 | 37,124 | 663,918 | 5.59 (5.54, 5.65) | 0.93 (0.92, 0.94) | 0.93 (0.92, 0.94) |
| 2009 | 36,687 | 659,807 | 5.56 (5.51, 5.62) | 0.93 (0.91, 0.94) | 0.92 (0.91, 0.94) |
| 2010 | 36,157 | 671,265 | 5.39 (5.33, 5.44) | 0.90 (0.88, 0.91) | 0.89 (0.88, 0.91) |
| 2011 | 36,472 | 675,075 | 5.40 (5.35, 5.46) | 0.90 (0.89, 0.91) | 0.89 (0.88, 0.91) |
| 2012 | 37,033 | 686,260 | 5.40 (5.34, 5.45) | 0.90 (0.89, 0.91) | 0.89 (0.88, 0.90) |
| **Age of mother, years** |  |  |  |  |  |
| Under 18 | 5,841 | 76,687 | 7.62 (7.43, 7.81) | 1.49 (1.45, 1.53) | 1.08 (1.05, 1.11) |
| 18-19 | 12,809 | 199,193 | 6.43 (6.32, 6.54) | 1.24 (1.22, 1.27) | 0.95 (0.93, 0.97) |
| 20-24 | 50,216 | 886,858 | 5.66 (5.61, 5.71) | 1.09 (1.08, 1.10) | 0.90 (0.89, 0.91) |
| 25-29 | 67,915 | 1,265,724 | 5.37 (5.33, 5.41) | 1.03 (1.02, 1.04) | 0.95 (0.94, 0.96) |
| 30-34 | 67,664 | 1,295,682 | 5.22 (5.18, 5.26) | 1.00 (Reference) | 1.00 (Reference) |
| 35-39 | 42,343 | 738,929 | 5.73 (5.68, 5.78) | 1.10 (1.09, 1.12) | 1.12 (1.10, 1.13) |
| 40 and over | 11,727 | 171,859 | 6.82 (6.71, 6.94) | 1.33 (1.30, 1.36) | 1.32 (1.29, 1.34) |
| **Deprivation quintile** |  |  |  |  |  |
| 1 (most deprived) | 81,999 | 1,261,026 | 6.50 (6.46, 6.55) | 1.44 (1.42, 1.45) | 1.34 (1.32, 1.36) |
| 2 | 60,280 | 1,031,727 | 5.84 (5.80, 5.89) | 1.28 (1.26, 1.30) | 1.24 (1.22, 1.25) |
| 3 | 45,309 | 862,178 | 5.26 (5.21, 5.30) | 1.15 (1.13, 1.16) | 1.12 (1.11, 1.14) |
| 4 | 38,126 | 771,250 | 4.94 (4.90, 4.99) | 1.07 (1.06, 1.09) | 1.07 (1.05, 1.08) |
| 5 (least deprived) | 32,801 | 708,751 | 4.63 (4.58, 4.68) | 1.00 (Reference) | 1.00 (Reference) |
| **Mother’s country of birth^c^** |  |  |  |  |  |
| UK | 199,357 | 3,507,324 | 5.68 (5.66, 5.71) | 1.00 (Reference) | 1.00 (Reference) |
| Non-UK | 59,141 | 1,127,465 | 5.25 (5.20, 5.29) | 0.92 (0.91, 0.93) | 0.89 (0.88, 0.90) |
| **Marital status/registration type** |  |  |  |  |  |
| Married | 124,912 | 2,499,063 | 5.00 (4.97, 5.03) | 1.00 (Reference) | 1.00 (Reference) |
| Joint registration/same address | 79,657 | 1,398,935 | 5.69 (5.66, 5.73) | 1.15 (1.14, 1.16) | 1.17 (1.16, 1.19) |
| Joint registration/different address | 32,143 | 450,500 | 7.13 (7.06, 7.21) | 1.47 (1.45, 1.48) | 1.43 (1.41, 1.45) |
| Sole registration | 21,803 | 286,434 | 7.61 (7.52, 7.71) | 1.57 (1.54, 1.59) | 1.51 (1.49, 1.54) |

^a^ Base model adjusted for baby’s sex and year of birth

^b^ Adjusted model adjusted for variables in Base model and additionally adjusted for age of mother, deprivation quintile, mother's country of birth (UK vs. non-UK) and marital status/registration type

^c^ Numbers based on study population excluding records with missing data on mother’s country of birth (n=143 in total)

**Table S2 The association between ethnic group, mother's country of birth, and preterm birth (singleton live births, England and Wales, 2006-2012)**

| **Ethnic group** | **Mother's country of birth** | **Live births** | **Preterm births** | **Preterm birth rate** | **Base model^a^** | **Adjusted model^b^** |
| --- | --- | --- | --- | --- | --- | --- |
|  |  | **N** | **n** | **% (95% CI)** | **OR (95% CI)** | **OR (95% CI)** |
| **White British** | UK | 2,891,589 | 161,341 | 5.58 (5.55, 5.61) | 1.00 (Reference) | 1.00 (Reference) |
|  | non-UK | 117,569 | 5,312 | 4.52 (4.40, 4.64) | 0.80 (0.78, 0.82) | 0.84 (0.82, 0.87) |
| **Other White** | UK | 67,614 | 3,585 | 5.30 (5.14, 5.47) | 0.94 (0.91, 0.97) | 0.96 (0.93, 0.99) |
|  | non-UK | 272,901 | 12,162 | 4.46 (4.38, 4.53) | 0.79 (0.78, 0.81) | 0.83 (0.81, 0.84) |
| **Indian** | UK | 45,137 | 2,891 | 6.40 (6.18, 6.63) | 1.16 (1.12, 1.21) | 1.29 (1.24, 1.34) |
|  | non-UK | 87,509 | 5,093 | 5.82 (5.67, 5.98) | 1.05 (1.02, 1.08) | 1.17 (1.14, 1.20) |
| **Pakistani** | UK | 66,860 | 4,407 | 6.59 (6.41, 6.78) | 1.19 (1.16, 1.23) | 1.26 (1.22, 1.30) |
|  | non-UK | 113,407 | 6,406 | 5.65 (5.52, 5.78) | 1.01 (0.99, 1.04) | 1.08 (1.05, 1.11) |
| **Bangladeshi** | UK | 13,704 | 888 | 6.48 (6.08, 6.90) | 1.18 (1.10, 1.26) | 1.23 (1.15, 1.32) |
|  | non-UK | 49,240 | 3,076 | 6.25 (6.04, 6.46) | 1.13 (1.09, 1.17) | 1.19 (1.14, 1.23) |
| **Black Caribbean** | UK | 30,061 | 2,524 | 8.40 (8.09, 8.72) | 1.55 (1.49, 1.62) | 1.26 (1.21, 1.31) |
|  | non-UK | 17,442 | 1,376 | 7.89 (7.50, 8.30) | 1.44 (1.37, 1.53) | 1.23 (1.16, 1.30) |
| **Black African** | UK | 11,206 | 762 | 6.80 (6.35, 7.28) | 1.23 (1.15, 1.33) | 1.11 (1.03, 1.20) |
|  | non-UK | 142,865 | 8,771 | 6.14 (6.02, 6.27) | 1.11 (1.08, 1.13) | 1.02 (1.00, 1.05) |
| **Mixed/Other** | UK | 169,499 | 10,764 | 6.35 (6.24, 6.47) | 1.15 (1.13, 1.17) | 1.06 (1.04, 1.08) |
|  | non-UK | 250,446 | 12,911 | 5.16 (5.07, 5.24) | 0.92 (0.90, 0.94) | 0.94 (0.93, 0.96) |
| **Not stated** | UK | 211,654 | 12,195 | 5.76 (5.66, 5.86) | 1.02 (1.00, 1.04) | 1.03 (1.01, 1.05) |
|  | non-UK | 76,086 | 4,034 | 5.30 (5.14, 5.46) | 0.94 (0.91, 0.97) | 0.98 (0.95, 1.01) |

^a^ Base model adjusted for baby’s sex and year of birth

^b^ Adjusted model adjusted for variables in Base model and additionally adjusted for age of mother, deprivation quintile, and marital status/registration type

**Table S3 The association between ethnic group and preterm birth (univariable analysis, singleton live births, England and Wales, 2006-2012)**

| **Ethnic group** | **Adjusted (base model)^a^** | **Adjusted (base+age of mother)^b^** | **Adjusted (base+** **deprivation quintile)^c^** | **Adjusted (base+UK vs. non-UK)^d^** | **Adjusted (base+reg type)^e^** |
| --- | --- | --- | --- | --- | --- |
|  | OR (95% CI) | OR (95% CI) | OR (95% CI) | OR (95% CI) | OR (95% CI) |
| **White British** | 1.00 (Reference) | 1.00 (Reference) | 1.00 (Reference) | 1.00 (Reference) | 1.00 (Reference) |
| **Other White** | 0.83 (0.82, 0.84) | 0.84 (0.83, 0.86) | 0.82 (0.81, 0.83) | 0.94 (0.92, 0.96) | 0.88 (0.86, 0.89) |
| **Indian** | 1.09 (1.07, 1.12) | 1.13 (1.10, 1.16) | 1.07 (1.04, 1.09) | 1.21 (1.18, 1.24) | 1.25 (1.22, 1.28) |
| **Pakistani** | 1.09 (1.07, 1.11) | 1.11 (1.09, 1.14) | 0.98 (0.96, 1.00) | 1.20 (1.17, 1.23) | 1.24 (1.22, 1.27) |
| **Bangladeshi** | 1.15 (1.11, 1.19) | 1.17 (1.13, 1.21) | 1.03 (0.99, 1.06) | 1.30 (1.26, 1.34) | 1.30 (1.26, 1.35) |
| **Black Caribbean** | 1.52 (1.47, 1.58) | 1.50 (1.45, 1.55) | 1.38 (1.34, 1.43) | 1.61 (1.55, 1.66) | 1.35 (1.30, 1.39) |
| **Black African** | 1.13 (1.10, 1.15) | 1.14 (1.12, 1.16) | 1.02 (1.00, 1.05) | 1.31 (1.28, 1.34) | 1.13 (1.11, 1.15) |
| **Mixed/Other** | 1.02 (1.01, 1.04) | 1.02 (1.01, 1.04) | 0.98 (0.96, 0.99) | 1.12 (1.10, 1.14) | 1.04 (1.03, 1.05) |
| **Not stated** | 1.00 (0.99, 1.02) | 1.01 (0.99, 1.03) | 1.00 (0.99, 1.02) | 1.04 (1.02, 1.06) | 1.03 (1.01, 1.05) |

^a^ Adjusted for baby’s sex and year of birth (base model)

^b^ Base model additionally adjusted for age of mother

^c^ Base model additionally adjusted for deprivation quintile

^d^ Base model additionally adjusted for mother's country of birth (UK vs. non-UK)

^e^ Base model additionally adjusted for marital status/registration type

**Table S4 Characteristics of the study population by mother’s country of birth (percentage, singleton live births, England and Wales, 2006-2012)**

| **Ethnic group** | **White British** | | **Other White** | | **Indian** | | **Pakistani** | | **Bangladeshi** | | **Black Caribbean** | | **Black African** | | **Mixed/Other** | | **Not stated** | |
| --- | --- | --- | --- | --- | --- | --- | --- | --- | --- | --- | --- | --- | --- | --- | --- | --- | --- | --- |
|  | **UK** | **non-UK** | **UK** | **non-UK** | **UK** | **non-UK** | **UK** | **non-UK** | **UK** | **non-UK** | **UK** | **non-UK** | **UK** | **non-UK** | **UK** | **non-UK** | **UK** | **non-UK** |
| **Baby's sex** |  |  |  |  |  |  |  |  |  |  |  |  |  |  |  |  |  |  |
| male | 51.3 | 51.2 | 51.4 | 51.5 | 51.0 | 51.5 | 51.0 | 51.0 | 51.1 | 50.5 | 50.4 | 51.5 | 51.2 | 50.5 | 51.4 | 51.4 | 51.3 | 51.5 |
| female | 48.7 | 48.8 | 48.6 | 48.5 | 49.1 | 48.5 | 49.0 | 49.0 | 48.9 | 49.5 | 49.6 | 48.5 | 48.8 | 49.5 | 48.6 | 48.6 | 48.7 | 48.5 |
| **Baby's year of birth** |  |  |  |  |  |  |  |  |  |  |  |  |  |  |  |  |  |  |
| 2006 | 13.5 | 14.2 | 18.0 | 8.6 | 12.5 | 12.0 | 13.1 | 13.2 | 11.3 | 14.0 | 14.3 | 15.3 | 14.1 | 12.9 | 12.5 | 12.2 | 22.6 | 19.4 |
| 2007 | 13.7 | 14.2 | 17.5 | 11.1 | 13.0 | 12.7 | 13.7 | 13.7 | 12.4 | 14.4 | 14.5 | 14.7 | 14.4 | 13.5 | 13.0 | 13.4 | 20.6 | 19.2 |
| 2008 | 14.0 | 14.2 | 15.0 | 12.9 | 13.0 | 13.0 | 14.5 | 14.7 | 13.3 | 14.2 | 14.1 | 14.7 | 14.2 | 14.2 | 13.7 | 13.8 | 19.8 | 20.2 |
| 2009 | 14.3 | 14.0 | 11.9 | 14.6 | 14.7 | 14.2 | 14.5 | 14.4 | 14.5 | 14.4 | 14.7 | 14.7 | 14.6 | 14.4 | 14.0 | 14.4 | 13.0 | 15.1 |
| 2010 | 14.8 | 15.0 | 12.3 | 16.5 | 15.2 | 15.1 | 14.2 | 13.9 | 15.0 | 14.2 | 14.7 | 13.4 | 14.4 | 15.1 | 14.7 | 15.0 | 9.0 | 9.9 |
| 2011 | 14.9 | 14.4 | 12.2 | 17.5 | 15.2 | 16.5 | 14.6 | 14.7 | 15.9 | 14.4 | 13.6 | 13.8 | 14.2 | 14.8 | 15.5 | 15.2 | 7.1 | 8.1 |
| 2012 | 14.9 | 14.1 | 13.1 | 18.8 | 16.5 | 16.6 | 15.4 | 15.5 | 17.7 | 14.4 | 14.2 | 13.4 | 14.1 | 15.2 | 16.6 | 16.0 | 7.9 | 8.0 |
| **Age of mother, years** | |  |  |  |  |  |  |  |  |  |  |  |  |  |  |  |  |  |
| Under 18 | 2.1 | 0.5 | 1.2 | 0.5 | 0.2 | 0.0 | 0.5 | 0.1 | 0.8 | 0.1 | 2.9 | 1.8 | 1.6 | 0.5 | 2.9 | 0.5 | 2.1 | 0.4 |
| 18-19 | 5.2 | 1.7 | 3.3 | 1.6 | 0.9 | 0.5 | 2.5 | 1.3 | 3.5 | 1.5 | 7.3 | 3.8 | 4.2 | 1.8 | 6.9 | 1.7 | 5.1 | 1.7 |
| 20-24 | 20.4 | 10.7 | 15.9 | 14.8 | 10.7 | 13.0 | 23.1 | 22.8 | 28.7 | 24.7 | 25.3 | 17.2 | 13.7 | 13.9 | 23.6 | 14.7 | 19.0 | 15.2 |
| 25-29 | 25.9 | 24.0 | 22.8 | 31.4 | 34.6 | 37.6 | 39.0 | 36.8 | 41.8 | 35.7 | 24.9 | 26.8 | 24.3 | 29.7 | 25.1 | 29.2 | 25.5 | 29.1 |
| 30-34 | 27.0 | 34.9 | 30.3 | 32.2 | 37.5 | 34.7 | 24.2 | 25.9 | 19.5 | 25.9 | 18.2 | 27.2 | 27.9 | 31.7 | 23.0 | 31.5 | 27.5 | 31.9 |
| 35-39 | 15.9 | 23.0 | 21.0 | 16.2 | 14.0 | 12.0 | 9.2 | 10.8 | 5.0 | 10.2 | 13.9 | 17.6 | 20.2 | 17.4 | 14.3 | 18.0 | 16.9 | 17.7 |
| 40 and over | 3.6 | 5.3 | 5.5 | 3.3 | 2.1 | 2.2 | 1.6 | 2.3 | 0.7 | 1.9 | 7.5 | 5.8 | 8.0 | 5.1 | 4.2 | 4.4 | 3.9 | 4.1 |
| **Deprivation quintile** | |  |  |  |  |  |  |  |  |  |  |  |  |  |  |  |  |  |
| 1 (most deprived) | 23.6 | 16.9 | 19.5 | 24.8 | 23.2 | 24.8 | 55.9 | 55.0 | 57.5 | 60.4 | 49.7 | 50.1 | 40.4 | 50.9 | 34.5 | 34.0 | 20.1 | 27.2 |
| 2 | 20.2 | 19.1 | 23.4 | 26.8 | 25.3 | 32.3 | 23.8 | 25.9 | 24.6 | 24.9 | 29.6 | 30.4 | 32.6 | 29.3 | 24.2 | 27.0 | 22.0 | 26.1 |
| 3 | 19.5 | 20.7 | 19.1 | 20.2 | 21.2 | 20.7 | 10.6 | 11.0 | 9.9 | 8.5 | 13.4 | 12.3 | 14.7 | 11.7 | 16.7 | 17.5 | 20.1 | 20.2 |
| 4 | 18.8 | 21.3 | 18.3 | 15.7 | 15.5 | 12.6 | 5.9 | 5.1 | 4.9 | 4.1 | 5.0 | 4.6 | 7.8 | 5.3 | 13.0 | 12.1 | 19.7 | 15.5 |
| 5 (least deprived) | 17.9 | 22.0 | 19.7 | 12.6 | 14.8 | 9.6 | 3.9 | 3.1 | 3.2 | 2.1 | 2.4 | 2.6 | 4.4 | 2.9 | 11.6 | 9.4 | 18.0 | 11.1 |
| **Marital status/ registration type** | | |  |  |  |  |  |  |  |  |  |  |  |  |  |  |  |  |
| Married | 45.3 | 67.0 | 60.4 | 65.9 | 93.4 | 97.6 | 92.7 | 97.5 | 86.0 | 97.0 | 21.1 | 35.8 | 54.7 | 61.8 | 38.5 | 77.1 | 49.5 | 78.3 |
| Joint registration / same address | 37.2 | 25.6 | 25.3 | 27.2 | 3.5 | 1.4 | 3.1 | 1.1 | 6.9 | 1.5 | 20.6 | 23.6 | 14.3 | 14.6 | 26.9 | 13.8 | 33.6 | 14.4 |
| Joint registration / different address | 11.2 | 4.2 | 8.4 | 2.9 | 1.7 | 0.4 | 2.2 | 0.5 | 4.3 | 0.8 | 37.0 | 23.5 | 18.3 | 11.9 | 20.6 | 4.9 | 10.0 | 3.5 |
| Sole registration | 6.4 | 3.2 | 5.8 | 4.1 | 1.5 | 0.6 | 2.0 | 0.9 | 2.8 | 0.7 | 21.3 | 17.1 | 12.7 | 11.7 | 14.0 | 4.3 | 6.9 | 3.9 |

^a^ Percentages based on study population excluding records with missing data on mother’s country of birth (n=143 in total)
